# 3D printing for advanced surgical planning in veterinary medicine—case studies, methods and future perspectives

**DOI:** 10.3389/fvets.2025.1596577

**Published:** 2025-07-09

**Authors:** Ramon Rodrigues de Lima, Lucas Rannier Ribeiro Antonino Carvalho

**Affiliations:** ^1^Department of Veterinary Medicine, University Center of João Pessoa—UNIPÊ, João Pessoa, Brazil; ^2^Department of Physiology and Pharmacology, Karolinska Institutet, Stockholm, Sweden

**Keywords:** 3D printing, dogs, veterinary medicine, surgery, preoperative planning

## Abstract

**Introduction:**

Conventional surgical planning in veterinary medicine is based on two-dimensional imaging, while advanced planning incorporates technologies such as three-dimensional reconstruction and virtual simulations. 3D printing has emerged as a promising tool, providing greater precision and customization of surgical procedures. The objective of the study is to analyze the applicability of 3D technology for surgical planning in veterinary medicine.

**Methods:**

The physical model is materialized using different techniques, such as stereolithography (SLA), selective laser melting (SLM), and fused deposition modeling (FDM), the latter being the most accessible and used in this report. To construct the digital models, the CT data are processed using inVesalius 3.1 software, a Brazilian program for segmentation and rendering of medical images. The resulting model is exported in .stl format and refined using Blender® software. The final printing is performed using the FDM method, using a slicer software, such as Ultimaker Cura®, which converts the 3D model into layers and generates commands for the printer. This process allows greater control over parameters such as temperature and speed, ensuring precision in the production of physical models.

**Results:**

Five different cases using 3D technology for surgical planning in dogs were described. In the first three cases, complete skulls were printed for oncological and temporomandibular joint (TMJ) surgery; in the last cases the areas of surgical interest were portions of the spinal column, the atlantoaxial region, and the thoracolumbar vertebrae.

**Conclusion:**

3D printing has been gaining ground in veterinary medicine, becoming a valuable tool in surgical planning and simulations. Even with the known relevance of new 3D technologies, more studies are needed in the development of new available materials, combination of techniques, accessibility, and medical education for the use of new applications and possibilities of 3D printing.

## Introduction—background

With the advancement of surgical techniques, the insertion of planning tools has proven essential to improve outcomes and reduce complications. Virtual surgical planning and 3D printing enable surgical planning and rehearsal that have been shown to improve surgical accuracy and decrease intraoperative time (1) thus allowing a more detailed understanding of the patient’s anatomy, enabling the choice of more appropriate and personalized approaches. Several studies have emphasized the importance of planning in veterinary surgeries, highlighting its contribution to reducing surgical time and improving postoperative outcomes (2).

Among technological innovations, advances in imaging techniques such as computed tomography (CT) and magnetic resonance imaging (MRI) allow the acquisition of detailed three-dimensional images, facilitating preoperative evaluation and procedure simulation (3, 4). At the same time, 3D technology has gained prominence in virtual and in-person planning of veterinary surgeries, providing an unprecedented level of precision (5, 6).

Virtual planning using specialized software allows the digital reconstruction of anatomical structures, enabling the simulation of different surgical techniques before the actual execution of the procedure (7). Using an additive manufacturing process, typically formed by layers of materials, either by the deposition of individual polymers or by the alternation of several different materials or cells (4).

Furthermore, 3D printing has been widely explored as a tool, allowing the creation of specific anatomical models for each patient (8), thus assisting in the visualization and manipulation of structures before surgery, which can contribute to reducing operative time and improving technical precision (5, 8).

In this context, the present study aims to describe five cases where 3D printing technology was used in veterinary surgical planning, addressing both virtual planning and the use of 3D printing for physical planning and simulations. The main advantages and limitations of these technologies will be discussed, seeking to contribute to the optimization of surgical practices in veterinary medicine.

## Methods

Three-dimensional printing (3D printing) refers to the process of creating objects from a digital model and is one of the processes under the umbrella of additive manufacturing (AM), a broader field. The virtual reference model can be generated by computer-aided design (CAD) software or by using 3D scanners to digitize the preexisting model or the patient’s own structure. However, for surgical planning, the used method is mainly based on medical scanning techniques such as magnetic resonance imaging (MRI) or computerized tomography (CT Scan) and subsequent reconstruction to a 3-dimensional file by a specific software (2, 9).

The materialization of the physical model is done through layer-by-layer production using different types of techniques and materials, for example: (1) stereolithography (SLA) which uses ultraviolet (UV) laser beams to selectively cure a photopolymer liquid (resin); (2) selective laser melting (SLM) which, similarly to SLA, uses a high-power laser that fuses metal powder particles into successive layers; and (3) fused deposition modeling (FDM) method, more accessible and widespread among the population (desktop 3D printers) which uses heated filaments extruded by the nozzle to form successive layers, which solidify when cooled (10, 11).

The raw data from the CT scan were used to construct the digital models described in this paper. The data were received from the veterinary team in DICOM format, an acronym for Digital Imaging and Communications in Medicine, an international standard for the exchange and storage of medical images and associated information (3, 4). After the validation, the set of data was uploaded to inVesalius 3.1 software for reconstruction of the virtual model. This free software was developed by Centro de Tecnologia da Informação Renato Archer (CTI), in Brazil and is used for visualization, segmentation and rendering of medical images from computed tomography and magnetic resonance (9).

The reconstruction software generates a three-dimensional file in “.stl” format (acronym for stereolithography), which is one of the most widely used for 3D printing and computer-aided design (CAD). This file is then imported into the modeling software for correction/cutting of the area of interest, removal of artifacts and image residues to generate the final version of the 3D virtual model. For 3D modeling in this paper, the software Blender® version 3.6 was used (12).

**Figure 1:**
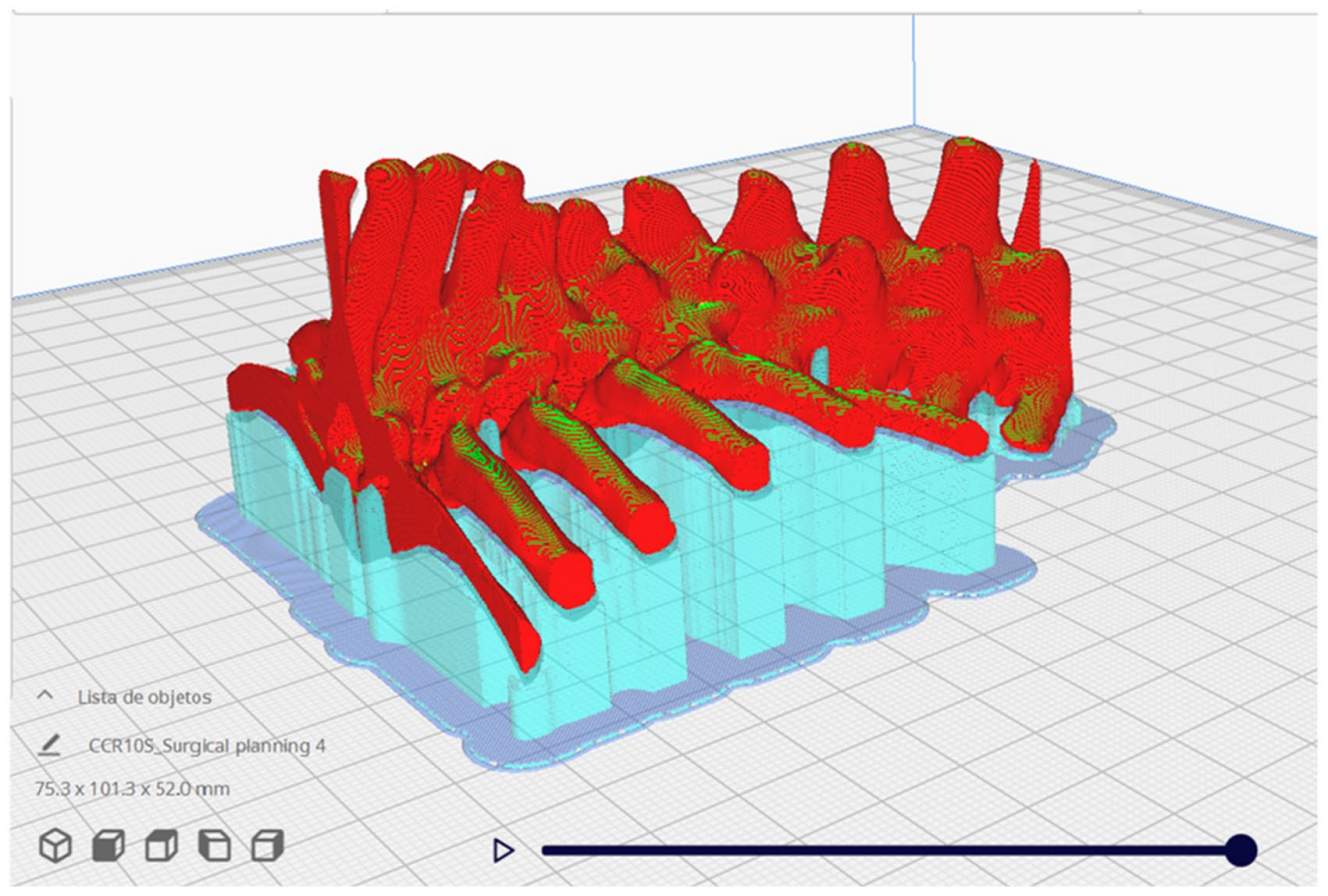
Screenshot of the Ultimaker Cura® 5.3.0 slicing software. In red, the 3D model of the phantom for printing, in light blue, the supports for printing and deposition of the filament layers, and in darker blue, the adhesion layers of the model on the printing table, known as the raft. Photo courtesy: 3D Medicine—3dmedicinebrasil@gmail.com.

The models were materialized using 3D printing through the FDM method, mentioned above. For this purpose, the virtual model was imported into the printing preparation software, called a “slicer,” as it transforms the three-dimensional file into slices or layers, and mathematically writes the commands so that the printer’s axes can move precisely during material deposition, thus creating the structures in three dimensions. Other parameters are also configured in the slicers, such as printing speed and temperature, layer height, number of walls, among others. By controlling the parameters and managing the printing commands, these software are known as the heart of the 3D printing workflow, for this paper the Ultimaker Cura® 5.3.0 (Ultimaker, Netherlands) slicer software was used (Figure 1) (13).

Unlike the printing of prostheses and other biomedical applications (10, 11), where material choice is crucial, the type of plastic used in surgical planning with FDM-printed models does not significantly impact the objectives. The critical point is the accuracy of the printed physical model compared to the virtual model from computed tomography; the planning model needs to reproduce the patient’s anatomical specifications as reliably as possible.

In this regard, some types of filaments are preferable to others; the printing quality needs to be high, but there is no need for high physical, chemical, or thermal resistance, which are relevant characteristics when choosing the material and its purpose.

For this work, we used polylactic acid (PLA) filaments, an organic material derived from renewable sources such as corn and sugarcane. PLA is widely used in 3D printing due to its easy handling, low melting temperature (around 190–220°C) and low warping rate, which makes it ideal for precise and high-quality prints (14). In addition, PLA has a smooth surface finish, with good adhesion between layers, characteristics of interest for a detailed model where every millimeter is significant.

After printing, the physical model was gently removed from the printing bed, the printing supports were removed, and the model was lightly finished with 80- to 120-grit sandpaper. The model measurements were checked for accuracy with the virtual reference model and then sent to the veterinarians responsible for the surgical procedures.

## Results

To demonstrate how 3D printing can be used as a complementary tool in advanced planning of surgeries in animals, five real cases were selected. All patients were dogs of different ages and surgical indications who were treated in 2024 by private veterinary clinics in João Pessoa, Brazil. The descriptions refer in particular to the use of 3D technology and the methods used to create the “phantom,” a physical or virtual model to simulate body parts, tissues or anatomical systems, which was used to support the surgical teams. For this reason, the surgical procedures and details of the interventions will not be discussed (Table 1).

**Table 1:** Use of 3D printing by FDM method for advanced surgical planning in dogs, description of the case series.

Case	Region of interest	Surgical procedure	Phantom size (mm)	Phantom weight (g)
1	Complete skull	Oncological surgery	180.0 × 134.5 × 110.6	156
2	Temporomandibular joint (TMJ)	Not reported	89.8 × 112.6 × 79.9	126
3	Complete skull	Oncological surgery	77.3 × 109.0 × 64.2	57
4	Cervical spine	Atlantoaxial fixation	50.9 × 48.9 × 37.8	13
5	Thoracic and lumbar spine	Spinal decompression	75.3 × 101.3 × 52.0	59

**Figure 2:**
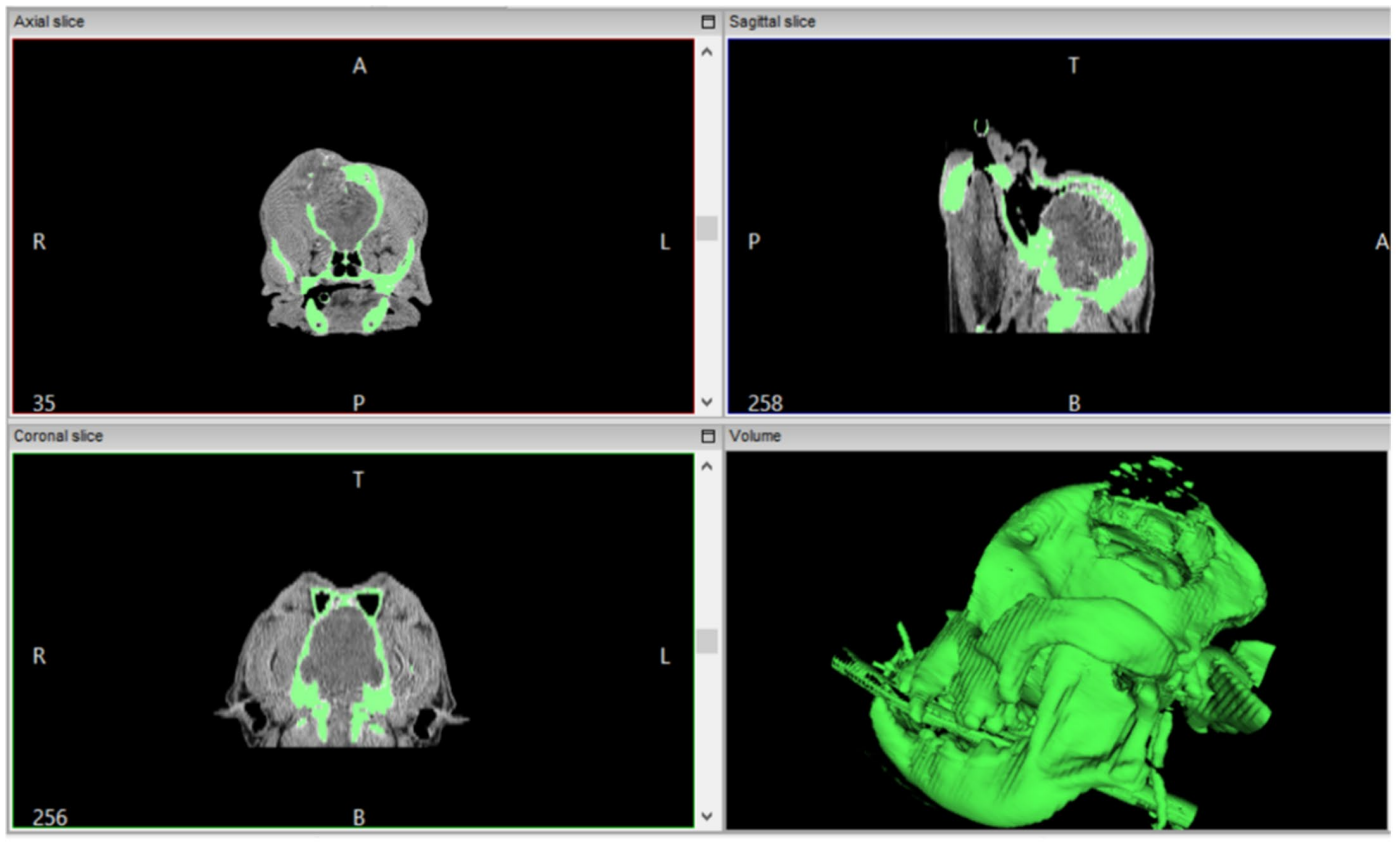
Screenshot of the InVesalius 3.1 software, used to reconstruct images obtained from computed tomography. In the upper portion, the axial and sagittal slices with the green markings of the regions of interest (bone) with threshold of 226 to 3,071. In the lower portion, the coronal slice, and the reconstructed 3D model (green) to be exported in .stl format.

For the first case, a six-year-old dog with a history of progressive volume increase in the region of the parietal and frontal bones on the left side, suggestive of neoplastic alteration, was referred for computed tomography. The data from the imaging exam (file in DICOM format) were sent for digital processing, to produce a phantom with the same anatomical characteristics, especially the areas with bone deformities. A set of 5 series totaling 208 images was received; the data were imported into the InVesalius 3.1 software for image reconstruction and production of the 3D surface, using a threshold for bone material selection of 226 to 3,071 (Figure 2).

**Figure 3:**
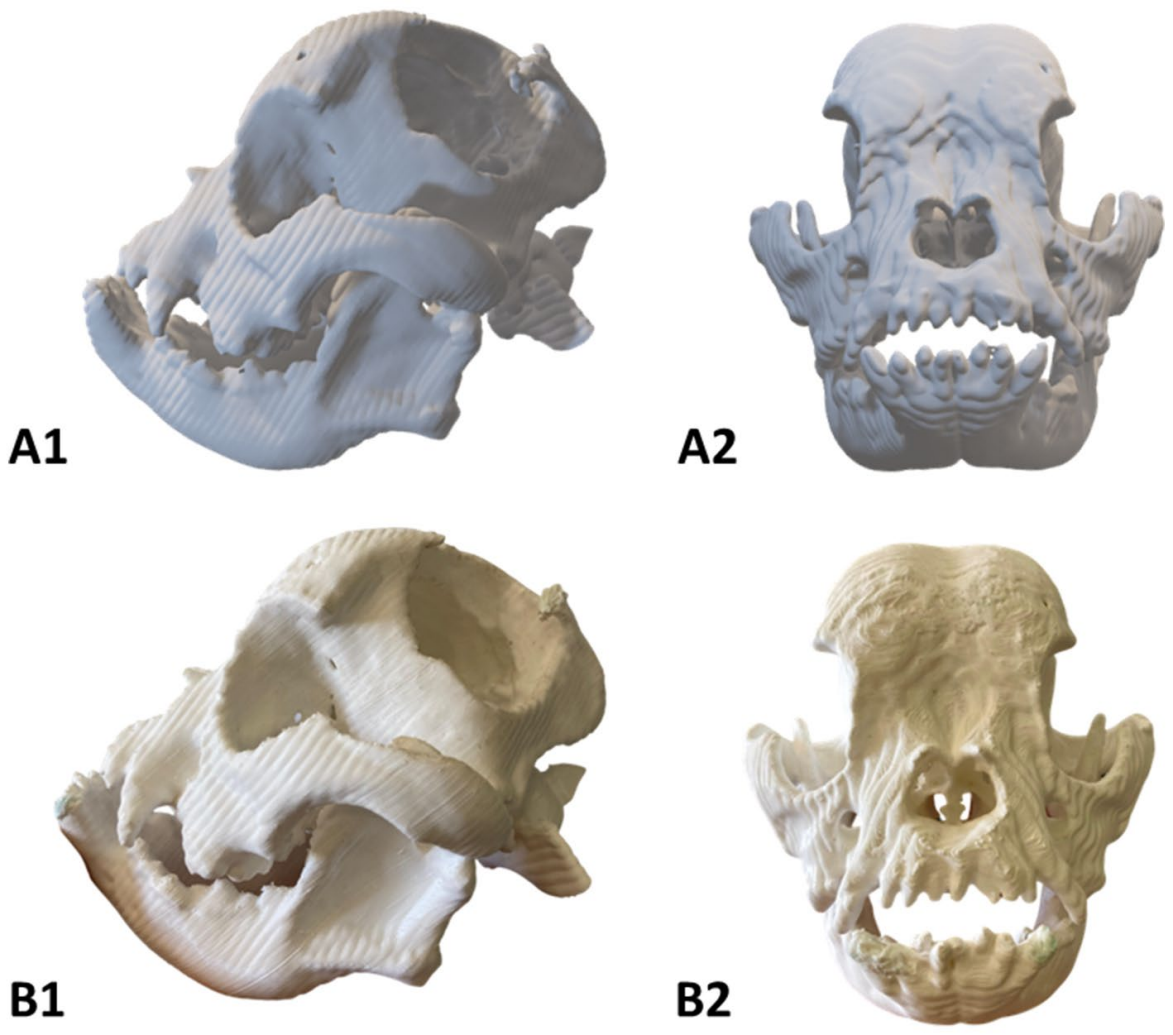
Phantom of a six-year-old dog with bone deformities in the head. **(A1-2)** Virtual model; **(B1-2)** Physical model. Photo courtesy: 3D Medicine—3dmedicinebrasil@gmail.com.

The 3D surface was exported in “.stl” format for modeling in Blender software, where image residues and artifacts were removed to isolate only the animal’s skull and jaw, thus composing the final virtual model (Figure 3A). This model can be used for surgical planning and simulations using specific software, but in this case, the file served as a basis for preparing the 3D printing of the physical phantom in the slicing software. The phantom was printed using white PLA filament, using a 0.4 mm extrusion nozzle with a layer height of 0.12 mm, a wall thickness of 1.6 mm with 25% infill in gyroid pattern, low speed and with tree supports (Figure 3B). At the end, the model was carefully removed from the printing table, the supports were removed, and the anatomical structures measured and compared with the digital file.

The physical phantom measured 180.0 × 134.5 × 110.6 millimeters (height × length × width) and weighed 156 grams (Figure 3B). The 3D printing was performed in collaboration with the Brazilian medical technology start-up ‘3D Medicine—3D Solutions for Health’. As expected, the structural bone changes on the left side of the skull were clearly visible on the 3D phantom, the floating particles observed on the CT Scan were removed (bone fragments), and the edges of the lesion presented the same anatomical consistency observed in the virtual file. This physical model served as a guide for advanced planning of the surgery, making it possible to simulate the surgical approach, discuss the anatomical structures related to the area of alteration, training, and communication between the team; however, it was not possible to obtain further information on the conduct of the procedure.

The second case described in Table 1 refers to a male dog with a history of temporomandibular joint alterations. The animal exhibited pain, along with difficulty in chewing and opening its mouth. The methodology for developing the phantom for surgical planning was the same as previously described; however, the computed tomography data were 4 series with 219 images and the reconstruction for the 3D model was using the threshold for bone material selection of 310 to 3,071. The virtual model was forwarded for selection of the area of interest and removal of the artifacts that remained after the density threshold filter. The final phantom measured 89.8 × 112.6 × 79.9 mm and was printed using PLA filament and same printing settings as case one described above (15% infill and 1.6 mm wall), the final phantom weighed 126 grams. All anatomical structures were reviewed and are in line with the imaging exam and the virtual model. No further details about the surgical procedure performed were provided.

**Figure 4:**
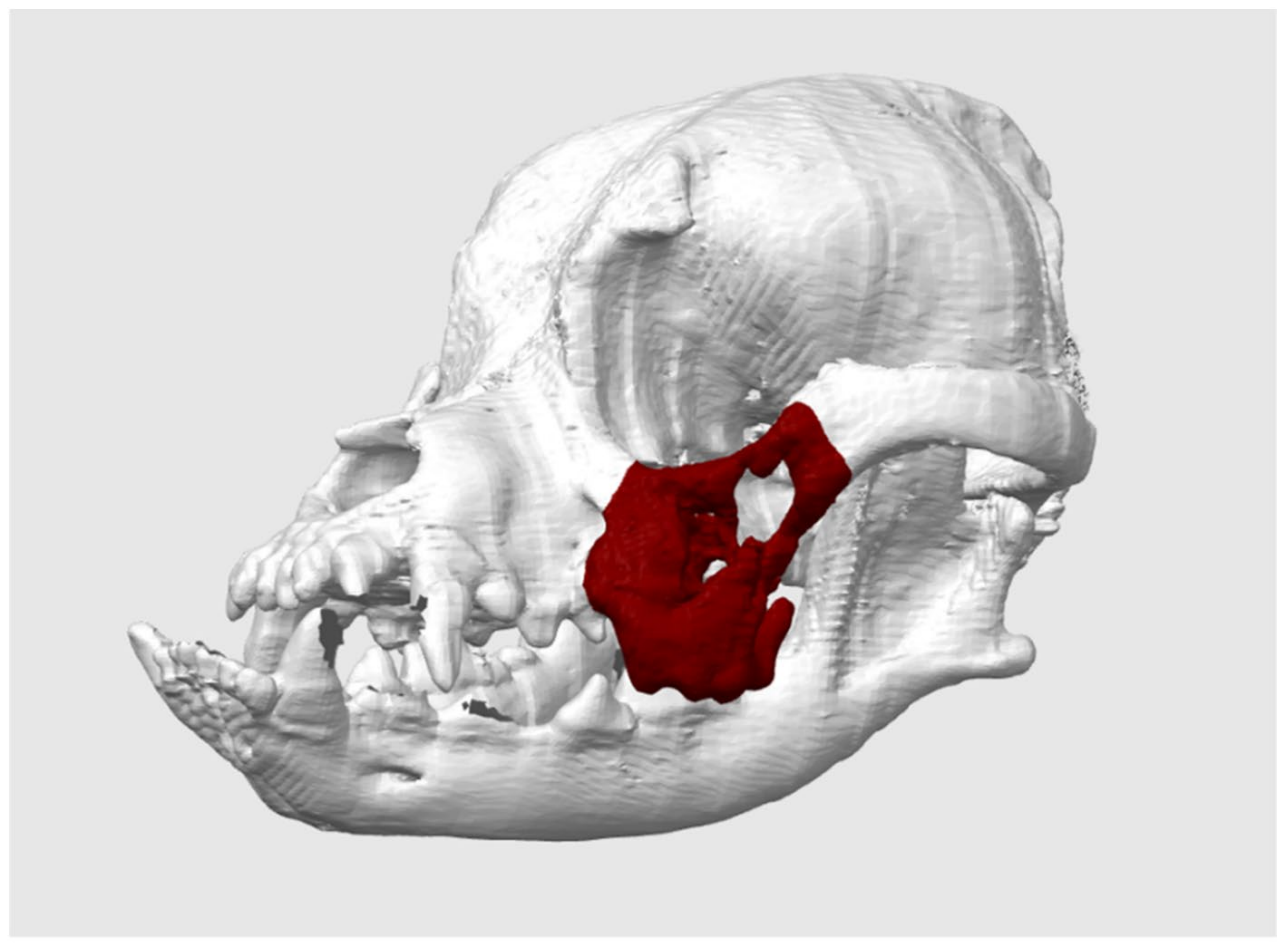
Phantom of a one-year-old dog with neoplastic bone alterations in the region of the zygomatic arch and left maxilla (virtually marked in red). Photo courtesy: 3D Medicine—3dmedicinebrasil@gmail.com.

The third case (Table 1) refers to a dog with neoplastic malformation in the region of the zygomatic arch and left maxilla. The animal presented intense pain, difficulties in chewing and no signs of ulceration. Using the methodology described above, the computed tomography data were 3 series with 468 images, the reconstruction for the 3D model was using the threshold for bone material selection of 226 to 3,071. The area of interest was marked in red as evidenced in Figure 4. The physical model was printed using the same settings described above, however for better accuracy a layer height of 0.12 mm was used.

**Figure 5:**
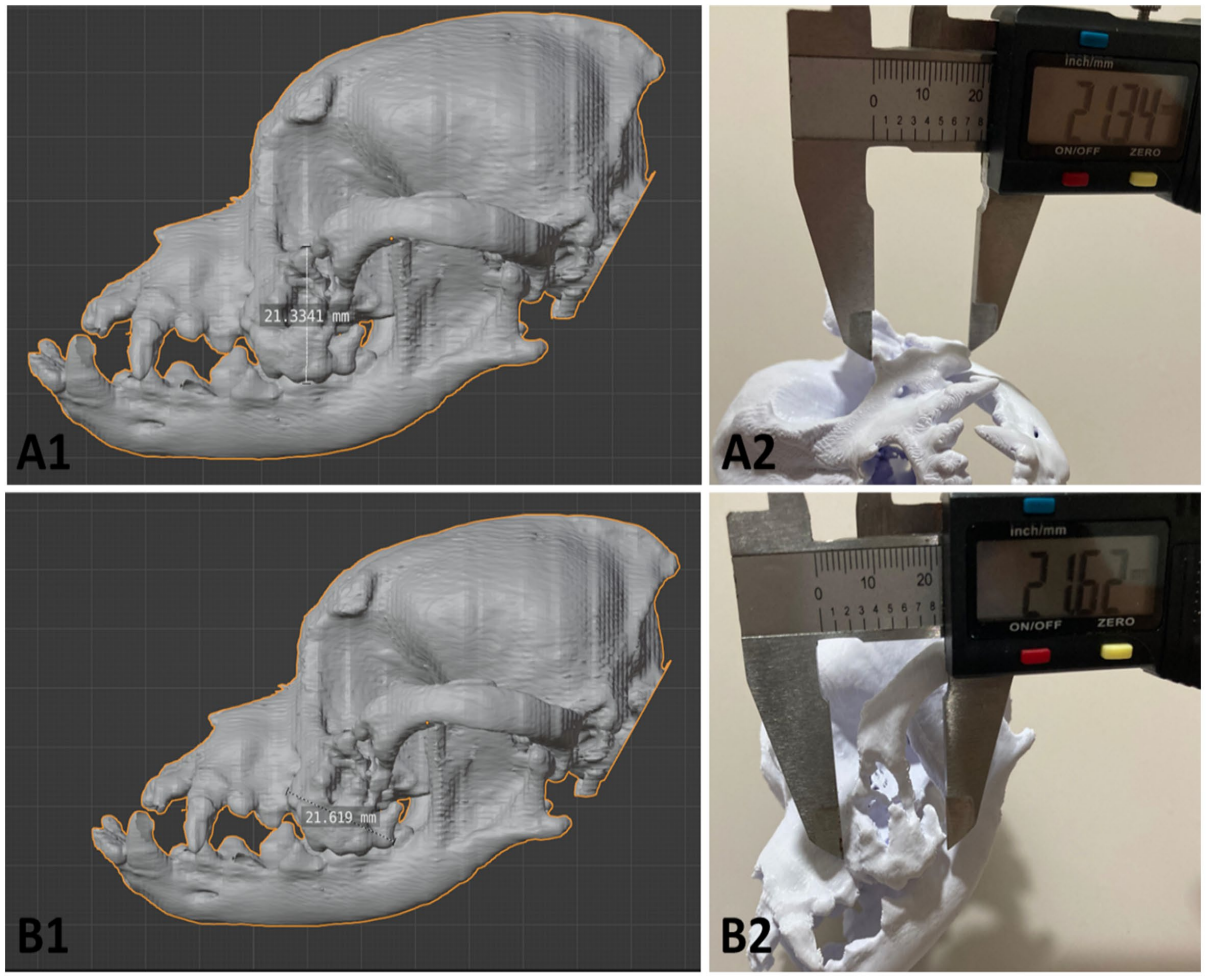
Records of the collection of measurements to investigate and demonstrate the accuracy of the 3D printing compared to the virtual model. **(A1,B1)** Screenshot of the 3D modeling software Blender, using the measurement tool to quantify the height and width of the margins of the neoplastic changes. **(A2,B2)** Photographic records of the measurements using calipers at the same reference points used in the digital model of the phantom. Photo courtesy: 3D Medicine—3dmedicinebrasil@gmail.com.

To demonstrate the accuracy of 3D printing, we compared the measurements of the physical phantom with those of the virtual model, using the Blender software measurement tools after calibration in millimeters, as well as a caliper. The data are presented in Table 2, and the following reference points were used: the height and width of the bone neoplasm margins in lateral view (points 1 and 2), the linear distance between the infraorbital foramina (point 3) and the linear distance between frontal bone edges (point 4), both measured in cranial view. The comparative analysis between the virtual and physical models revealed excellent dimensional accuracy, with a maximum absolute error margin of 0.1145 mm and a relative error margin of less than 0.4% in all measurements performed. Figure 5 illustrates the measurements obtained from both the virtual model and the 3D printed phantom.

**Table 2:** Comparison between virtual and 3D printed physical measurements of anatomopathological structures for surgical planning.

Reference points	Virtual model (mm)	3D printed model (mm)	Absolute difference (mm)	Percentage error (%)
Tumor height	21.3341	21.34	0.0059	0.0276
Tumor width	21.6190	21.62	0.0010	0.0046
Linear distance between the infraorbital foramina	29.8345	29.72	0.1145	0.3839
Linear distance between frontal bone edges	27.2178	27.14	0.0778	0.2857

**Figure 6:**
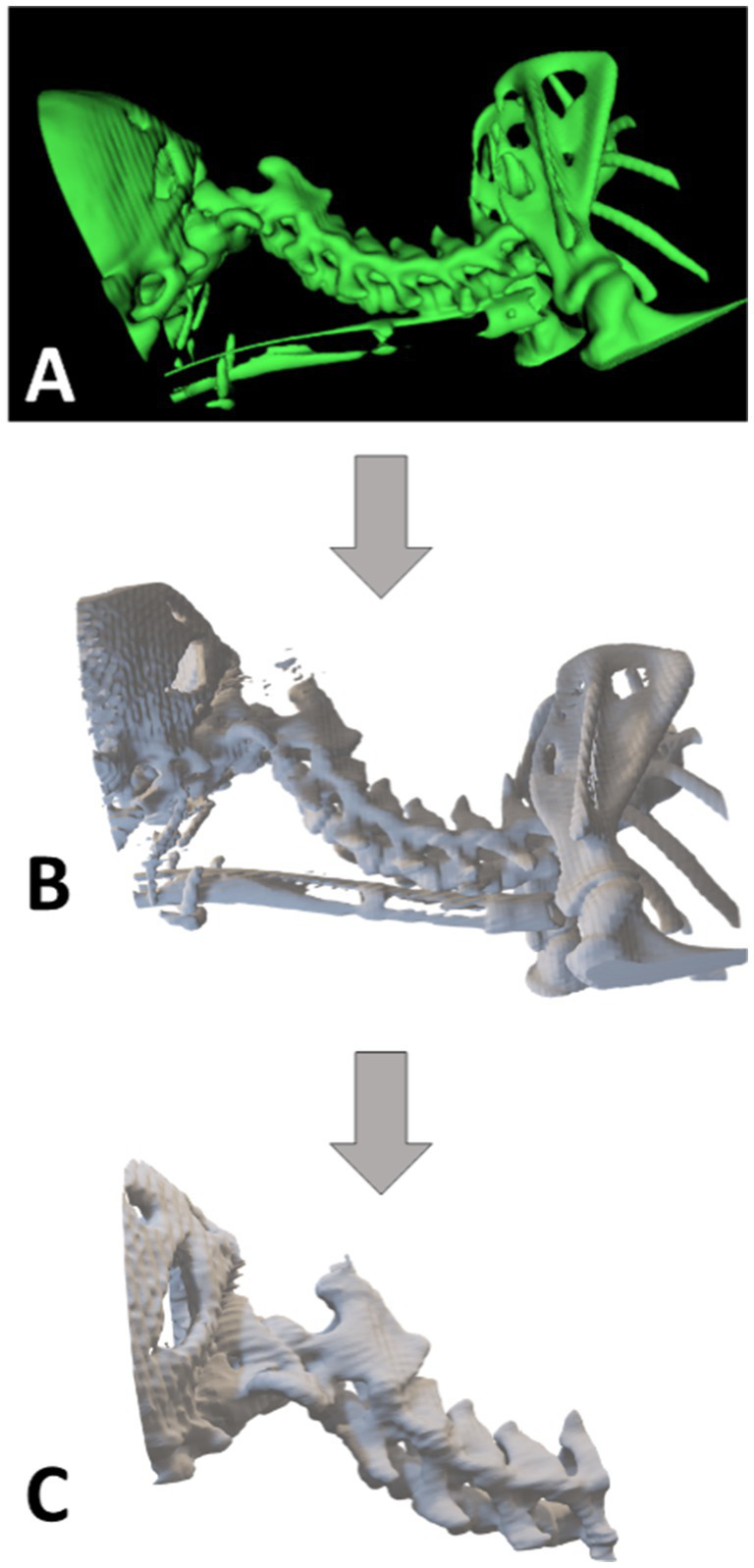
Roadmap for printing the Yorkshire Terrier puppy phantom; **(A)** Rendering of the CT scan data—InVersalius software; **(B)** 3D modeling and adjustments of the area of interest—Blender software; **(C)** Final virtual model sent for 3D printing through the slicing software—Ultimaker Cura. Photo courtesy: 3D Medicine—3dmedicinebrasil@gmail.com.

The following two cases were for planning surgical interventions in the spinal column (Table 1—cases 4 and 5), a problem with increasing casuistry in small animal veterinary medicine nowadays. The third case was of a Yorkshire terrier puppy with a history of intense pain in the cervical region, head rotation and neurological alterations. After computed tomography and diagnosis of atlantoaxial dislocation, surgery to fix the atlantoaxial joint was indicated. The imaging data received to produce the 3D phantom were 3 series with 192 images in total. The files were reconstructed from the occipital region of the skull to the first thoracic vertebrae, with a limit for selection of bone material from 226 to 2,464. The virtual model generated after reconstruction was imported into the Blender modeling software where artifacts were removed, and the area of interest was selected (Figure 6A). The final phantom measured 50.9 × 48.9 × 37.8 mm with delicate support areas and thin walls, which pose a challenge for FDM 3D printing. For this reason, a 0.2 mm extrusion nozzle, tree support structures, 100% infill and a significantly slower print time of 40 mm/s ensure a small but accurate model was printed. After printing and removing the supports, the phantom weighed 13 grams and contained all the anatomical structures of interest so that the veterinary team could plan the procedure (Figure 6B).

**Figure 7:**
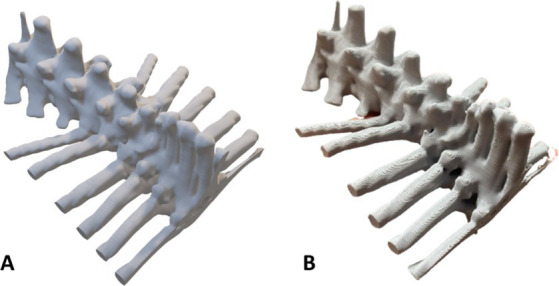
Phantom of a 2-year-old male dog with vertebral compression in the thoracolumbar transition (T12-L2). **(A)** Virtual model; **(B)** Physical model. Photo courtesy: 3D Medicine—3dmedicinebrasil@gmail.com.

The last case (5) was a 2-year-old male dog, mixed breed, whose region of interest was the thoracolumbar transition (T12-L2) due to severe vertebral compression. Two series of 366 images in total were received from the computed tomography for reconstruction of the phantom. After rendering the images, the file was exported to the modeling software where the region of interest (ROI) was delimited. For this surgical planning, the phantom was modeled with a cranial and caudal margin of interest in addition to a cranial portion of the last ribs to serve as reference points for the physical model. The final model measured 75.3 × 101.3 × 52.0 mm and was printed using PLA filament with a layer height of 0.12 mm, 0.4 mm extrusion nozzle, 100% infill, with tree-type supports and low speed. After printing and removing the supports, the phantom weighed 59 grams with the desired anatomical precision for surgical planning (Figure 7).

## Discussion

Conventional surgical planning is commonly based on clinical and diagnostic information, anatomical studies, and analysis of two-dimensional imaging exams, such as radiographs and computed tomography scans. Advanced surgical planning, in addition to conventional techniques, incorporates the use of cutting-edge technologies for three-dimensional reconstruction of the surgical target (area, organ, tissue, anatomical structure), either virtually or physically, customization of guides and implants tailored to the patient (precision medicine), and simulations of the surgical plan in virtual and augmented reality environments (10, 15).

The benefits of the 3D printing method for surgical planning are promising for veterinary medicine, and can be used in different areas of application. One of the possibilities is in managing the visualization and understanding of the animal’s anatomy with greater clarity and the veterinarian’s practice before surgery, as it allows the production of a replica of the unique anatomy of the patient’s region of interest, allowing examination and manipulation before surgery, improving the understanding of complex anatomical structures, characteristics resulting from malformations, neoplasias or lesions (3, 4, 16).

Altwal (4) emphasizes that these impressions allow surgeons to adapt their surgical plans to the patient’s anatomy and rehearse the procedures. These preoperative procedural rehearsals are specific to each patient and aim to reduce intraoperative decisions, duration of surgery, exposure to intraoperative radiation, if necessary, and especially preoperative planning for complex surgeries, deformities and fractures. Thus, the veterinarian can predict possible anatomical differences, reducing the margin of error and ensuring the animal’s safety only by preoperative planning (16, 17).

Scheuermann (6) discusses the application of 3D technology in minimally invasive plate osteosynthesis (MIPO) surgeries, where there was a 34-min reduction in surgical time in the group of animals that used 3D printing when compared to the group that did not use it. The 34-min reduction in surgery duration is potentially advantageous, as each minute of reduction in surgical time was seen to have a reduction in the likelihood of developing infection at the surgical site. This reduction in surgical time can be attributed to the efficient indirect fracture reduction, as well as the application of a plate that was anatomically shaped for 3D printing, thus demonstrating a reduction in surgical time and cost (16).

Furthermore, Memarian (2) discusses the importance of this technology in client communication and education, demonstrating that physical models increase recipient satisfaction and aid communication by providing a better visual understanding of the actual medical condition, pathology, and surgical procedure that must be performed on the animal and explained to the pet owner. These prototypes lead to improved communication with the client by allowing owners to better understand the surgical complexity, possible complications, and the cost associated with the procedures. For example, in corrective osteotomy surgeries, biomodels have been found to be very effective for owner education, providing a visual representation of both the angular deformity of the limb and the intended correction. Furthermore, 3D printed models have been incorporated into both clinical communication and the surgical environment, increasing the learning curve of the professional team by establishing an in-house production of individual models for surgical guidance and surgical cutting guides (1, 3, 16).

The costs for this procedure are still under debate at this time, but it is likely that it would result in a decrease in surgery time and anesthesia time, and therefore costs may be similar to current standards of care, but with greater safety (18).

While the cost of printing may seem expensive, it is crucial to consider the diverse range of materials available. Depending on the intended purpose of the printed model, there are more affordable alternatives that can be explored, such as standard resin. The key is to select materials that align with specific requirements, ultimately optimizing cost and functionality. However, it is expected that as technology continues to advance, the cost of production will steadily decrease, and therefore 3D printing will become more economically viable for both veterinarians and patient owners (3, 19).

Furthermore, the reduction in production costs is directly reflected in the final price of the procedures, making them more accessible to a larger portion of the population. This aspect is particularly relevant in countries such as Brazil, where, despite the high demand (19).

## Conclusion

3D printing represents a significant advance for veterinary medicine, providing greater precision, safety, and efficiency in surgical planning. Despite the initial challenges related to cost, the trend is that the evolution of technology will make its application increasingly accessible. Hence the importance of conducting more studies so that this technology can be incorporated continuously. It is expected that 3D printing will become a standard tool, benefiting both professionals and guardians and their animals, ensuring better clinical outcomes.

## Data Availability

The datasets presented in this article are not readily available because the datasets are 3D models of patients and cannot be shared. For more information about 3D modeling and printing for veterinary medicine, or how to print the 3D model for surgical planning, please contact 3dmedicinebrasil@gmail.com.
